# Predicting the Compressive Strength of the Cement-Fly Ash–Slag Ternary Concrete Using the Firefly Algorithm (FA) and Random Forest (RF) Hybrid Machine-Learning Method

**DOI:** 10.3390/ma15124193

**Published:** 2022-06-13

**Authors:** Jiandong Huang, Mohanad Muayad Sabri Sabri, Dmitrii Vladimirovich Ulrikh, Mahmood Ahmad, Kifayah Abood Mohammed Alsaffar

**Affiliations:** 1 School of Mines, China University of Mining and Technology, Xuzhou 221116, China; 2 Peter the Great St. Petersburg Polytechnic University, 195251 St. Petersburg, Russia; mohanad.m.sabri@gmail.com; 3 Department of Urban Planning, Engineering Networks and Systems, Institute of Architecture and Construction, South Ural State University, 76, Lenin Prospect, 454080 Chelyabinsk, Russia; ulrikhdv@susu.ru; 4 Department of Civil Engineering, University of Engineering and Technology Peshawar (Bannu Campus), Bannu 28100, Pakistan; ahmadm@uetpeshawar.edu.pk; 5 University of Mashreq, Baghdad 10023, Iraq; kiffaya_alsaffar@yahoo.com

**Keywords:** hybrid machine-learning method, concrete, compressive strength

## Abstract

Concrete is the most widely used material in construction. It has the characteristics of strong plasticity, good economy, high safety, and good durability. As a kind of structural material, concrete must have sufficient strength to resist various loads. At the same time, due to the brittleness of concrete, compressive strength is the most important mechanical property of concrete. To solve the disadvantages of the low efficiency of the traditional concrete compressive strength prediction methods, this study proposes a firefly algorithm (FA) and random forest (RF) hybrid machine-learning method to predict the compressive strength of concrete. First, a database is built based on the data of published articles. The dataset in the database contains eight input variables (cement, blast furnace slag, fly ash, water, superplasticizer, coarse aggregate, fine aggregate, and age) and one output variable (concrete compressive strength). Then, the correlation of the eight input variables was analyzed, and the results showed that there was no high correlation between the input variables; thus, they could be used as input variables to predict the compressive strength of concrete. Next, this study used the FA algorithm to optimize the hyperparameters of RF to obtain better hyperparameters. Finally, we verified that the FA and RF hybrid machine-learning model proposed in this study can predict the compressive strength of concrete with high accuracy by analyzing the R values and RSME values of the training set and test set and comparing the predicted value and actual value of the training set and test machine.

## 1. Introduction

Concrete is made up of cementitious material, aggregate, water, admixture, and mineral admixture following a certain proportion by uniform mixing, compaction molding, curing hardening, and becoming a kind of artificial stone [1,2,3,4,5]. It is one of the most important civil building materials at present [6,7,8,9,10,11,12,13]. Concrete not only has the characteristics of abundant raw materials, low price, and a simple manufacturing process but also has the characteristics of high compressive strength and good durability [6,14,15,16,17]. Due to these characteristics, concrete has been widely used in construction, shipbuilding, the machinery industry, and other fields [18,19,20,21]. 

However, in the process of concrete preparation, when cement particles contact with water, the clinker minerals on the surface of cement particles will immediately hydrolyze or hydrate with water to generate new hydration products and release a certain heat, which is called the hydration reaction of concrete [22,23]. For concrete engineering, the cement and water hydration reaction needs to release a certain amount of heat, called the hydration heat of concrete [24,25]. 

The heat release rate and size of the hydration heat mainly depend on the mineral composition of cement, cement fineness, admixture, and other factors, among which the mineral composition is the most important factor [26]. The rate and quantity of the hydrating heat releases of tricalcium aluminate, tricalcium silicate, and dicalcium silicate decrease successively. The cement commonly used in concrete is Portland cement. After mixing Portland cement, there are mainly tricalcium silicate, dicalcium silicate, tricalcium aluminate, and iron solid solutions, which react with water. The chemical equation involved in the hydration process of cement concrete is as follows:

(1) $$3\left(CaO\cdot SiO_{2}\right)+6H_{2}O=3CaO\cdot 2SiO_{2}\cdot 3H_{2}O+3Ca{\left(OH\right)}_{2}$$

(2) $$2\left(CaO\cdot SiO_{2}\right)+4H_{2}O=3CaO\cdot 2SiO_{2}\cdot 3H_{2}O+3Ca{\left(OH\right)}_{2}$$

(3) $$3CaO\cdot Al_{2}O_{3}+6H_{2}O=3CaO\cdot Al_{2}O_{3}\cdot 6H_{2}O$$

(4) $$4CaO\cdot Al_{2}O_{3}\cdot Fe_{2}O_{3}+7H_{2}O=3CaO\cdot Al_{2}O_{3}\cdot 6H_{2}O+CaO\cdot Fe_{2}O_{3}\cdot H_{2}O$$

Although the hydration heat can accelerate the growth of the early strength of concrete, increase the frost resistance of concrete, and has the advantage for concrete construction projects in the winter, the heat in the concrete due to hydration gathered in the interior is not easy to release, which results in a dramatic increase in the temperature inside the concrete, and thus a large temperature difference between the concrete inside and outside, and this causes apparent temperature stress and makes the concrete crack, which greatly affects the strength and other properties of concrete [27]. 

At the same time, the cement production process will discharge emissions, which has brought a high burden to the environment [23]. To ease the preparation of concrete with the cement hydration heat, concrete compressive strength, and other properties of the impact of the cement production process and the burden of carbon emissions to the environment, using fly ash, blast furnace slag, metakoalin, and other mineral admixtures to replace part of the cement as supplementary cementing materials has become the main solution.

Researchers have successfully applied fly ash into concrete after a great deal of research and achieved good results in improving the performance of concrete. Zhu et al. studied the influence of fly ash on the durability of concrete pavement, and the research results showed that the concrete mixed with fly ash could not only reduce the amount of cement but also have a good influence on the durability of concrete [1,28]. Huang et al. studied the effect on the properties of mixed concrete with the expansive agent and fly ash, the research results showed that the incorporation of fly ash on concrete when adding an expansive agent or not has a different influence on the durability of concrete, fly ash has little influence on the dilatancy of concrete without the expansive agent, and fly ash has a positive impact on the durability of concrete with the expansive agent. 

When the content of fly ash exceeds 10%, this effect is weakened, and when the expansion agent and fly ash are mixed into concrete at the same time, the original fly ash must be used [29]. Jang et al. studied the influence of the water–binder ratio and fly ash content on concrete durability, and the research results showed that the compressive strength of concrete with fly ash was better than the compressive strength of concrete without fly ash, and the durability of concrete improved with the increase in the fly ash content. 

Blast furnace slag is a kind of organic melting mixture discharged from a blast furnace when smelting pig iron, and it is a type of industrial waste residue. In recent years, increasing researchers have paid attention to the improvement of concrete performance by blast furnace slag. Vibha et al. studied the effects of blast furnace slag on the slump, compressive strength, bending strength, and splitting tensile strength of concrete at different displacement levels [30]. Wang et al. studied the influence of air-warming blast furnace slag and crushed limestone on the mechanical properties of concrete. 

The results show that air-warming blast furnace slag and crushed limestone both have positive effects on the mechanical properties of concrete, and the influence of air-warming blast furnace slag on the mechanical properties of concrete is better than that of crushed limestone [31]. The compressive strength of concrete is one of the most important properties. However, there are relatively few studies on the influence of fly ash and blast furnace slag on the compressive strength of concrete. The use of fly ash and blast furnace slag in improving the concrete compressive strength performance and alleviate the impact of carbon emissions on the environment both play an important role. However, the prerequisite for mineral admixtures to play a role in concrete is that the quality of mineral admixtures must meet the requirements. 

Otherwise, the addition of mineral admixtures will not only not improve the compressive strength of concrete but also negatively affect the properties, such as cohesion, segregation, and condensation time of secreting water. As the quality of fly ash and blast furnace slag is different in different regions, it is of great significance to study the influence of fly ash and blast furnace slag on the compressive strength of concrete. The laboratory test method is the most common method used by researchers to study the compressive strength of concrete. 

However, the laboratory experiment method has the disadvantages of low efficiency, high cost, and energy consumption. To solve these problems, many researchers have proposed the machine-learning method to predict the compressive strength of concrete. Cui et al. proposed the XGBoost model based on the Boosting Tree algorithm to predict the compressive strength of concrete, and the research results show that the model has high prediction accuracy when the compressive strength is greater than 40 MPa [32]. 

Al-Shamir et al. proposed a regularized extreme learning machine (RELM) to predict the compressive strength of concrete and used k-fold cross-validation to evaluate the reliability of the established RELM model. The results show that compared with other models, the RELM model can predict the compressive strength of concrete more accurately [33]. The above machine-learning models have achieved good results in predicting the compressive strength of concrete, which confirms the feasibility of using machine-learning models to predict the compressive strength of concrete.

Although there are many scholars put forward using machine-learning method to predict the compressive strength of concrete, it should be pointed out that most of the prediction models still exist problems, such as low efficiency and bad prediction results [34,35,36,37,38,39,40,41,42,43,44,45,46,47,48,49]. Thus, looking for a more efficient and precise machine-learning model to predict the compressive strength of concrete is necessary [50,51,52,53,54]. This study aims to propose a new hybrid model to accurately and effectively predict the compressive strength of cement-fly ash-slag ternary concrete. 

A new regression technique RF algorithm has been used to predict the compressive strength, which is more accurate and computationally efficient than other commonly used prediction tools [55,56,57,58,59,60,61]. Nonparametric stochastic forest is a set of stochastic decision trees used to deal with nonlinear regression problems, and its superiority in numerical prediction has been proved by several civil engineering problems. However, the feasibility of estimating the compressive strength of cement-fly ash-slag ternary concrete by RF method has not been studied yet. 

In addition, RF can measure the relative importance score of each input variable to quantify the significance level of each influence parameter on the compressive strength of concrete. Therefore, to improve the accuracy and efficiency of the machine-learning model to predict the compressive strength of concrete, a method of predicting concrete compressive strength with the FA and RF hybrid machine-learning model is proposed in this paper.

## 2. Methodology

### 2.1. Dataset Collection

The database is the basis for verifying whether a machine-learning model can accurately predict the compressive strength of concrete. To ensure the sufficient accuracy of the data sets, 225 data sets were collected from the previous literature published by other authors, and a database was established with these data sets [62]. The data sets in this database all have a common feature, namely, they all contain cement, blast furnace slag, fly ash, water, superplasticizer, coarse aggregate and fine aggregate, age, and concrete compressive strength variables. The input variables are cement, blast furnace slag, fly ash, water, superplasticizer, coarse aggregate, fine aggregate, and age. The output variable is concrete compressive strength. 

A reliable database is a key to verifying the prediction accuracy of concrete compressive strength by the model. To verify the reliability of the database, the author analyzed the data set of the database as shown in Table 1. Table 1 analyzed the maximum value, median, mode, mean, standard deviation, and variance of the nine variables. According to Table 1, the range of cement, blast furnace slag, fly ash, water, superplasticizer, coarse aggregate, fine aggregate, age, and concrete compressive are included in the database are 132–491 g, 11–214 g, 24.5–195 g, 121.8–247 g, 1.7–22.19 g, 814–1080.8 g, 612–880 g, 3–100 days, and 7.32–76.44 MPa, respectively. The mode values of these nine variables are 446, 24, 141, 162, 6, 967, 801, 28, and 27.68, respectively.

**Figure 1:**
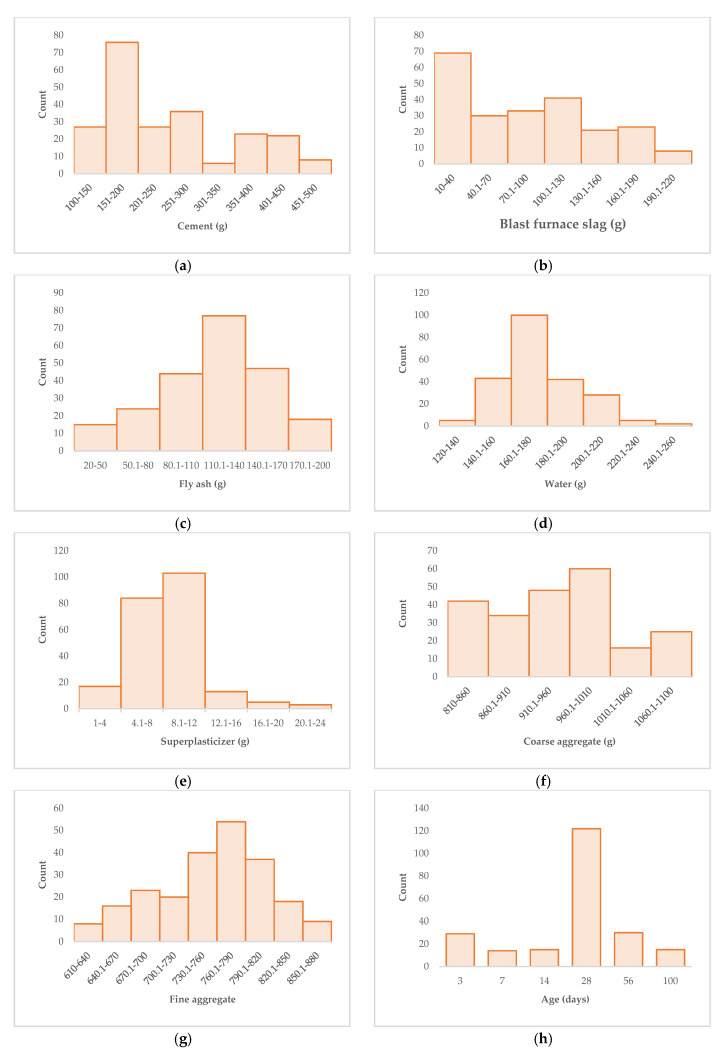

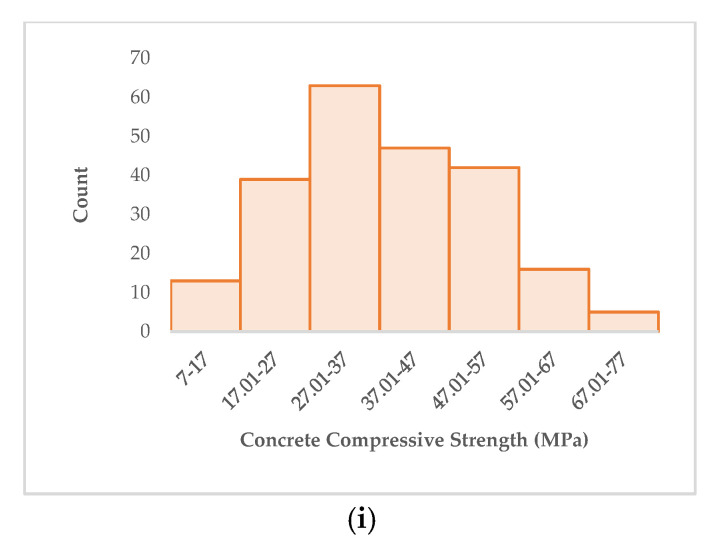
Frequency distribution histogram of variables. (**a**) Cement; (**b**) Blast furnace slag; (**c**) Fly ash; (**d**) Water; (**e**) Superplasticizer; (**f**) Coarse aggregate; (**g**) Fine aggregate; (**h**) Age; (**i**) Concrete compressive strength.

To see the data distribution of the nine variables in the database more intuitively, we made the frequency distribution histogram of the nine variables, as shown in Figure 1. Figure 1 clearly shows that the frequency distribution histogram of fly ash, water, superplasticizer, age, and concrete compressive strength is unimodal. The frequency distribution histograms of blast furnace slag and coarse aggregate are single-peak type, while the frequency distribution histograms of cement and fine aggregate are double-peak. In other words, the data of the nine variables in the database are reasonably distributed and cover a wide range. Therefore, it can achieve a better result in predicting the compressive strength of concrete with the data set in the database.

**Figure 2:**
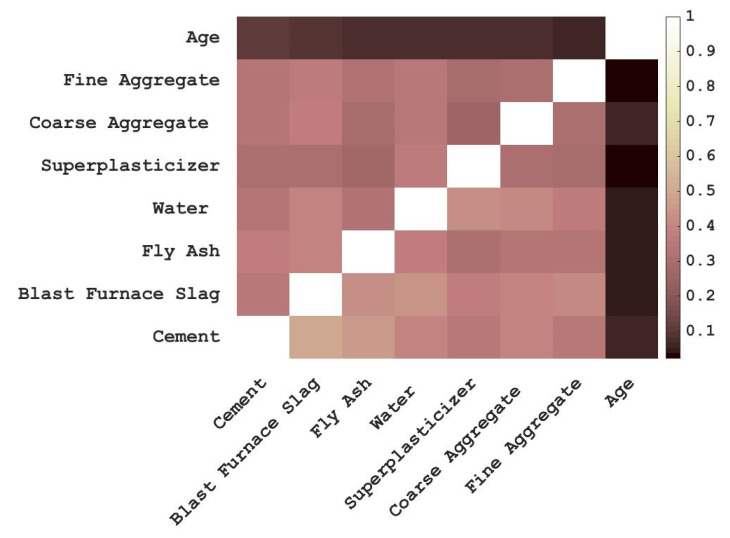
Correlation coefficients matrix diagram.

The analysis of two or more variables that are correlated is called correlation analysis, which measures the closeness of the relationship between two variables. The correlation coefficient between input variables is high positive or high negative, in other words, the high correlation between input variables will affect the prediction effect of the model. To determine the correlation between input variables, this study conducted correlation analysis on the eight input variables before the model training the result is shown in Figure 2. It can be seen from Figure 2 that there is a certain correlation among the eight input variables, among which the correlation between Cement and Blast Furnace Slag is about 0.5 at the highest; however, the correlation between them is less than 0.6. That is to say, using the eight variables as the input variables to predict the compressive strength of concrete the model will not be affected by multicollinearity.

### 2.2. Applied Machine-Learning Models

In this study, a hybrid FA and RF machine-learning model is proposed to predict the compressive strength of concrete, where FA is used to tune the hyperparameters of RF and RF is used to predict the compressive strength of concrete.

FA is a meta-heuristic algorithm based on firefly flashing behavior. The main idea of the firefly algorithm is that a firefly with weak light moves to a firefly with strong light to complete a position update. The firefly algorithm has the following three assumptions: (1) fireflies are not differentiated in gender, and fireflies with strong luminescence will attract fireflies with weak luminescence. (2) The attraction between fireflies is proportional to the brightness. (3) The brightest fireflies make random movements. Based on the above three assumptions, the mathematical model of the firefly algorithm is obtained as follows (Appendix A):

First, *I_i_* is used to represent the absolute brightness of the *i*th firefly, and the objective function value of the firefly’s location was expressed by the absolute brightness of the firefly. The absolute brightness *I_i_* of the firefly located at $\vec{x}\left(x_{i1},x_{i2}\cdots x_{id}\right)$ is equal to the function value at $\vec{x_{i}}$ —that is, $I_{i}=f\left({\vec{x}}_{i}\right)$.

If the *i*th firefly is brighter than the *j*th firefly, the *j*th firefly will move toward the *i*th firefly due to the attraction of the *i*th firefly. The attraction of the *i*th firefly to the *j*th firefly is proportional to its relative brightness. The relative brightness of the *i*th firefly to the *j*th firefly is defined as:

(5) $$I_{ij}\left(r_{ij}\right)=I_{i}e^{-\gamma r_{ij}^{2}}$$

where *I_i_* is the brightness of the *i*th firefly, and *γ* is the absorption coefficient of light intensity.

Assuming that the attraction of the *i*th firefly to the *j*th firefly is proportional to the brightness of the *i*th firefly to the *j*th firefly, the attraction of the *i*th firefly to the *j*th firefly is defined as:

(6) $$\beta_{ij}\left(r_{ij}\right)=\beta_{0}e^{-\gamma r_{ij}^{2}}$$

where *β*_0_ is the maximum attraction, *β_ij_* is the attraction of the *i*th firefly to the *j*th firefly, *r_ij_* is the Cartesian distance of the *i*th firefly to the *j*th firefly, and is defined as:

(7) $$r_{ij}=\|x_{i}-x_{j}\|=\sqrt{\sum_{k=1}^{d}{\left(x_{i,k}-x_{j,k}\right)}^{2}}$$

Suppose that the position of the *j*th firefly is updated because it is attracted by the *i*th firefly, and the updated formula is:

(8) $$x_{j}\left(t+1\right)=x_{j}\left(t\right)+\beta_{ij}\left(r_{ij}\right)\left(x_{i}\left(t\right)-x_{j}\left(t\right)\right)+\alpha \xi_{j}$$

where *t* is the number of iterations, $\vec{\xi }$ is the random number vector, *α* is constant, and usually *α* ∈ [0, 1]. As can be seen from the position update formula, position update mainly depends on the attractiveness, if *β*_0_ = 0 in the position update formula, the formula becomes a random walk model. The code of the firefly algorithm can be found in the appendix section. 

**Figure 3:**
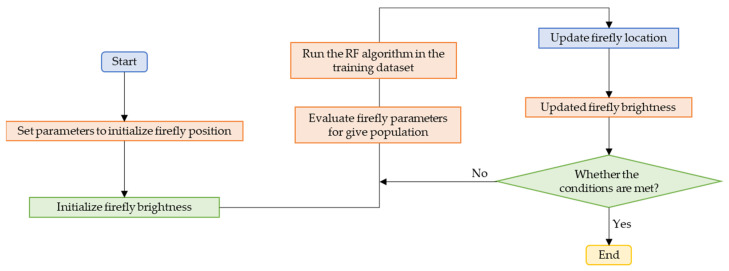
Flow chart of the hybrid model.

Figure 3 gives the flow chart of the hybrid model using FA and RF. First, the collected data sets are randomly divided into training data sets and test data sets. Then, FA is used to tune the rf’s hyperparameters, and the performance of the RF is evaluated using a 10-fold cross-validation model. Once the optimal hyperparameters are determined, the RF model is used to predict the compressive strength of the test data set.

**Figure 4:**
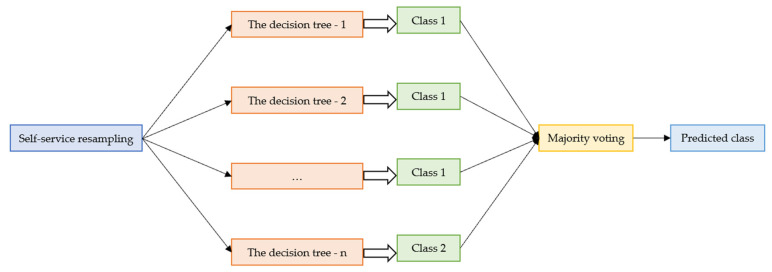
Flow chart of random forests.

RF algorithm is a typical integrated learning algorithm. RF is based on statistical theory and uses autonomous resampling technology to extract multiple sample sets from training samples. The algorithm constructs several decision tree models by using the extracted sample sets and gathers the decision tree models together to obtain the final result by voting or taking the average. RF is an ensemble classifier constructed by a decision tree model $\{h\left(X,\theta_{k}\right),k=1,\cdots K\}$ using bagging integration, where $\{\theta_{k}\}$ is an independent random vector with the same distribution. Input sample *x* into RF and find the final output $f\left(x\right)=majority\{h\left(x,\theta_{k}\right)|k=1,2,\cdots K\}$. RF can classify a variety of data accurately. It can process a large number of input parameters and evaluate the importance of variables in determining categories. The construction process of the RF algorithm is shown in Figure 4.

## 3. Results and Discussion

### 3.1. Hyperparameter Tuning

**Figure 5:**
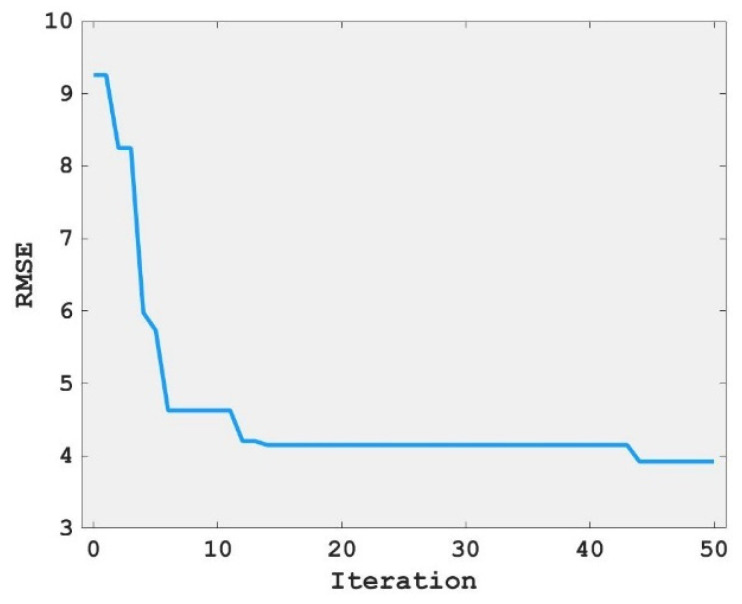
Relationship between the iteration and RSME value.

Machine learning has its super-parameters in operation, and these parameters have a great influence on itself. These parameters are often not obtained by training but need to be set before the learning process begins. There are many hyperparameters in the machine-learning model. Before running the machine model, the hyperparameters can be optimized utilizing hyperparameter tuning to improve the performance of the machine-learning model. In this study, the FA model was used to optimize the hyperparameter of the RF model. The relationship between iterations and RSME value is shown in Figure 5. It can be seen from Figure 5 that with the increase in iteration times, the RSME value decreases sharply at first and then tends to be stable. The variation trend shows that the hyperparameter tuning of the RF model with FA can achieve good results.

**Figure 6:**
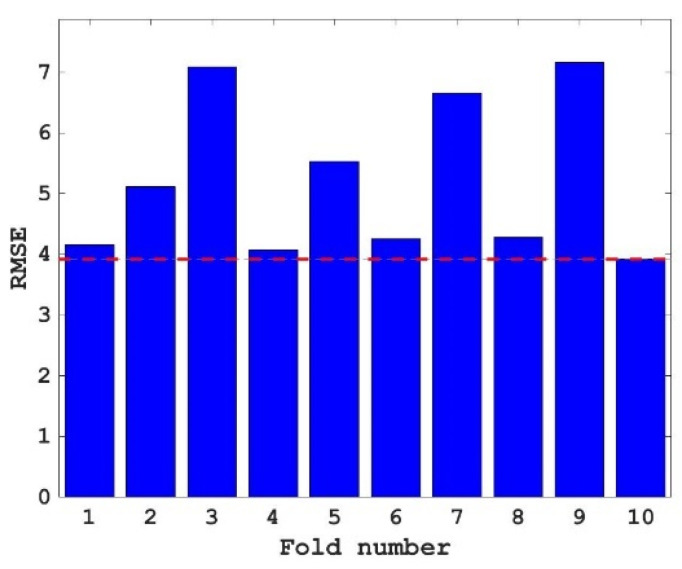
RMSE values of different folds.

A common test method used to test the accuracy of an algorithm is 10-fold cross-validation. The main idea of this method is to randomly divide the data set into ten parts, and take nine of them and one of them as the test data, in turn, for the experiment. To further obtain the corresponding optimized hyperparameters, 10-fold cross-validation was used for hyperparameter tuning in this study. The results of the 10-fold cross-validation are shown in Figure 6. It can be seen from Figure 6 that the minimum value of RSME is obtained at the 10th fold, which is about 3.9 (as shown by the red dotted line), and the results show that there will be no over-fitting phenomenon in predicting the compressive strength of concrete with the proposed RF model.

### 3.2. Model Evaluation

In the field of machine learning, models need to be evaluated to verify the performance of trained models. Different types of models use different evaluation methods. After the establishment of the machine-learning model for concrete compressive strength prediction, the next important step is to evaluate the prediction effect of the established machine-learning model. In this study, the accuracy of the prediction of concrete compressive strength by the RF model was verified by comparing the predicted value and actual value of concrete compressive strength. 

**Figure 7:**
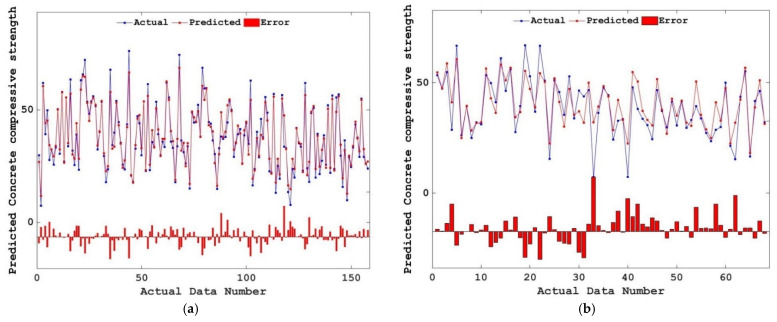
Comparison of the actual compressive strength and predicted compressive strength. (**a**) Training set; (**b**) Testing set.

Figure 7 shows the comparison between the predicted value and the actual value of concrete compressive strength in the training set and test set, where the horizontal line represents the error. It can be seen from Figure 7 that the predicted value of the training set has a high consistency with the actual value. Although the predicted value of the test set has several electrical points with large errors from the actual value, the predicted value is generally consistent with the actual value. The results show that the RF model can accurately predict the compressive strength of concrete.

**Figure 8:**
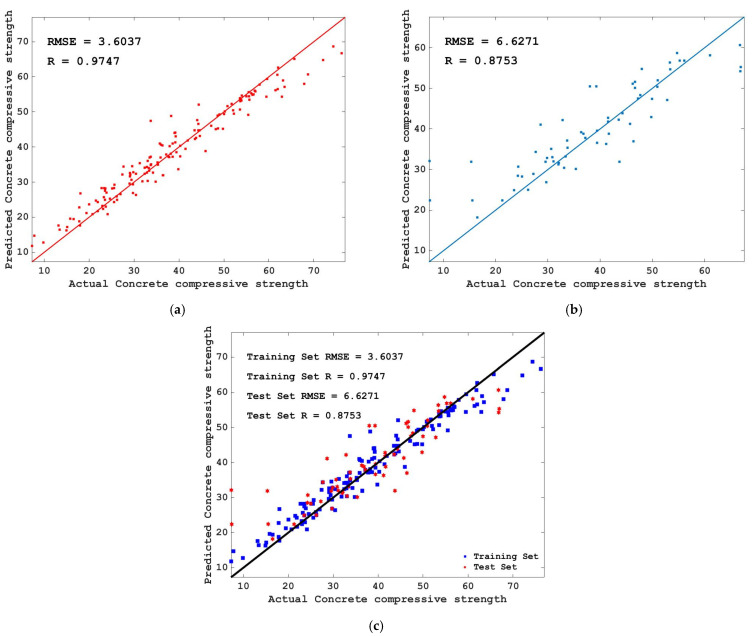
Comparison of predicted value and actual value of the data set. (**a**) Training set; (**b**) Testing set; (**c**) Training set and testing set.

To see the fitting effect of predicted value and actual value of training set and test set more intuitively, the scatter diagram of predicted value and actual value of training set and test set is given in Figure 8. It can be seen from Figure 8 that the concrete compressive strength of both the training set and the test set is concentrated at 0–70 MPa, and the predicted value and actual value of the training set and the test set have a good fitting effect on the whole, with only a few points with large errors in the test set. In the training set, there were several points where the actual value of concrete compressive strength was about 6 MPa, and the predicted value was as high as 20 or 30 MPa. 

However, it should be noted that the minor differences in individual data points do not affect the overall predictive performance of the RF model, that is, the RF model can accurately predict the compressive strength of concrete. The R value of the training set is 0.9747, the RSME value is 3.6037, the R value of the test set is 0.8753, and the RMSE value is 6.6271. Thus, the R value and RMSE value of the training set and the test set have common characteristics—namely, their R value is high, and their RSME value is low. It is proved again that the RF model tuned by FA has a good effect on predicting the compressive strength of concrete, and there is no over-fitting situation.

### 3.3. Variable Importance Evaluation

**Figure 9:**
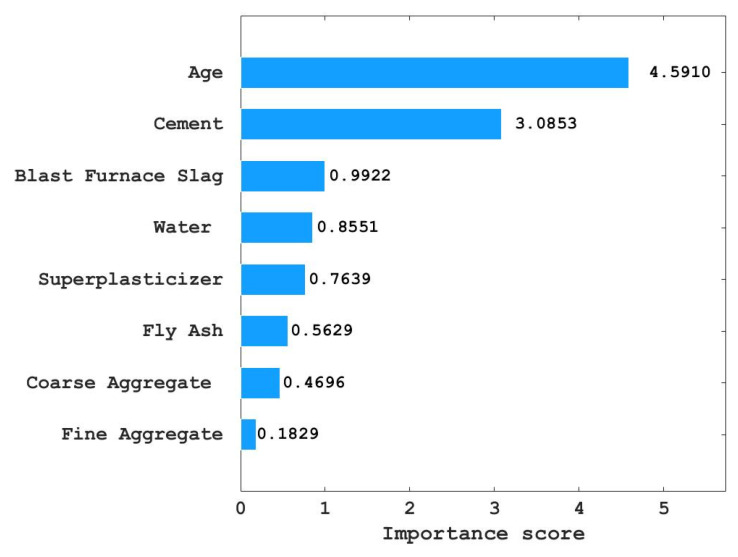
Variable importance of the compressive strength.

Figure 9 shows the importance scores of the eight input variables to the compressive strength of concrete obtained by the RF model. It can be seen from Figure 9 that age has the highest score of 4.5910 among the eight variables—that is to say, age has the greatest influence on the compressive strength of concrete among the eight input variables, and the compressive strength of concrete is proportional to age. Thus, the compressive strength of concrete increases with the increase in age within a certain range. The importance of cement to concrete compressive strength scored is 3.0853—the second-highest among the eight input variables. 

That is to say, cement also has a great influence on the compressive strength of concrete, and the compressive strength of concrete is proportional to the amount of cement. The importance of blast furnace slag, water, superplasticizer, fly ash, coarse aggregate, and fine aggregate to the compressive strength of concrete is 0.8551, 0.7639, 0.5629, 0.4696, 0.1839, decreasing successively. Thus, the importance of these six variables to the compressive strength of concrete decreases successively. 

From the importance score of variables, it can be seen that the compressive strength of concrete is proportional to the eight variables, that is, the increase in any one of the eight variables will improve the compressive strength of concrete. Since age and cement have a great influence on the compressive strength of concrete, engineers should pay more attention to the age and cement when designing concrete with high compressive strength, and less attention should be paid to the amount of fine aggregate.

## 4. Conclusions

In this study, a hybrid FA and RF machine-learning model was proposed to predict the compressive strength of concrete. A database of 225 data sets was established based on previously published articles, which was used as the data set for predicting the compressive strength of concrete. The data set took cement, blast furnace slag, fly ash, water, superplasticizer, coarse aggregate, fine aggregate, and age as the input variables. The concrete compressive strength was used as the output variable. The FA algorithm was used to tune the hyperparameters of the RF algorithm, and then the results of the hyperparameter tuning were verified by 10-fold cross-validation. Finally, the accuracy of the model was verified by analyzing the R value and RSME values well as the predicted value and actual value of the training set and the test set. The following conclusions can be drawn from the research process.

1. Using FA to tune the hyperparameter of RF, the RSME value decreases greatly at first and then tends to be stable with the increase in iteration number, this proves that FA can achieve better results in adjusting the hyperparameter optimization of the RF model, which is better than the random selection of hyperparameters.
2. The RF model tuned by FA can be used to predict the compressive strength of concrete and achieve better results. The R values of the training set and the test set were 0.9747 and 0.8753, respectively, and the RSME values were 3.6037 and 6.6271, respectively—that is, the training set and the test set both had high R values and low RSME values, and the consistency between the predicted value and the actual value of the concrete compressive strength of the training set and the test set was high. The above two conclusions prove that the FA and RF mixed models achieved better results in predicting the compressive strength of concrete.
3. The importance scores of age, cement, blast furnace slag, water, superplasticizer, fly ash, coarse aggregate, and fine aggregate to the compressive strength of concrete decreased successively and were all positive. That is, the compressive strength of concrete was proportional to these eight variables, and the importance of these eight variables to the compressive strength of concrete decreased in turn.

## Figures and Tables

**Table 1:** Variable data analysis.

Variables	Minimum	Maximum	Median	Mode	Average	Std.	Variance
Cement (kg/m^3^)	132	491	213.8	446	446	106.2	1127.82
Blast furnace slag (kg/m^3^)	11	214	97	24	24	58.28	3388.44
Fly ash (kg/m^3^)	24.5	195	122	141	141	38.5	1479.09
Water (kg/m^3^)	121.8	247	175.1	162	162	21.26	451.99
Superplasticizer (kg/m^3^)	1.7	22.1	8.4	6	6	3.46	11.98
Coarse aggregate (kg/m^3^)	814	1080.8	942	967	967	78.46	5156.35
Fine aggregate (kg/m^3^)	612	880	764.4	764.4	801	58.23	3391.26
Age (days)	3	100	28	28	28	23.71	561.76
Compressive strength (MPa)	7.32	76.24	36.44	36.44	27.68	14.19	201.32

## Data Availability

The data presented in this study are available on request from the corresponding author.

## Appendix A

**Algorithm A1** Code of Firefly Algorithm
**begin** Objective function f(x), x = (x_1_, ..., x_d_) ^T^ Generate initial population of fireflies x_i_ (i = 1, 2, ..., n) Light intensity I_i_ at x_i_ is determined by f(x_i_) Define light absorption coefficient γ **while** (t<MaxGeneration) **for** i = 1 : n all n fireflies **for** j = 1 : i all n fireflies **if** (I_j_ > I_i_) Move firefly i towards j in d-dimension via Levy flights **end if** Attractiveness varies with distance r via exp[−γr] Evaluate new solutions and update light intensity **end for** j **end for** i Rank the fireflies and find the current best **end while** Postprocess results and visualization **end**
